# Extracts of Common Vegetables Inhibit the Growth of Ovary Cancer Cells

**DOI:** 10.3390/foods11162518

**Published:** 2022-08-20

**Authors:** Paulina Furdak, Natalia Pieńkowska, Grzegorz Bartosz, Izabela Sadowska-Bartosz

**Affiliations:** Laboratory of Analytical Biochemistry, Institute of Food Technology and Nutrition, College of Natural Sciences, University of Rzeszow, 4 Zelwerowicza Street, 35-601 Rzeszow, Poland

**Keywords:** ovary cancer, SKOV-3, PEO1, cytotoxicity, polyphenols, horseradish, garlic, horseradish, curly kale, tea

## Abstract

There is recent interest in a diet that can be recommended for patients suffering from cancer. In this respect, the effects were studied of the extracts of several common fruits, herbs and vegetables on the viability of two human ovary cancer cell lines (SKOV-3 and PEO1) in vitro. Normal human MRC-5 fibroblasts were used as a control cell line. The extracts of garlic, horseradish and curly kale as well as green and black tea were the most effective in lowering the viability of ovarian cancer cells, while not affecting the viability of MRC-5 fibroblasts. Except for garlic and horseradish, the cytotoxic effects of the extracts correlated with their polyphenol content. The examination of changes in the content of ATP and glutathione, in the level of reactive oxygen species, mitochondrial potential and mitochondrial mass did not show a consistent pattern, suggesting that various extracts may act via different mechanisms. Although the extracts’ toxicity to cells in vitro is a first and direct suggestion concerning their possible anticancer effects in vivo, these results point to potential vegetable candidates to become diet components recommended for ovary cancer patients.

## 1. Introduction

There are many considerations concerning a diet that can be recommended for the prevention of cancer and for patients suffering from cancer. No decreased susceptibility to breast, colorectal, and prostate cancer was found in vegetarians compared to non-vegetarians, although a lower risk of colorectal cancer was associated with a semi-vegetarian diet and a pesco-vegetarian diet compared to a non-vegetarian diet [1]. A diet rich in lignans and isoflavonoids has been proposed to lower the propensity for breast cancer [2]. A restricted calorie diet, reducing the growth of experimental tumors, was demonstrated to be mediated through the toxic effect of ketone bodies on cancer cells [3]. A fasting mimicking diet was found to have a positive effect on the chemotherapy in patients with HER2-negative stage II/III breast cancer and to curtail chemotherapy-induced DNA damage in the T-lymphocytes of these patients [4]. The Ketogenic Diet, a high-fat/low-carbohydrate/adequate protein diet, was recently proposed as an adjuvant therapy in cancer treatment [5,6,7]. Lycopene-rich diets and green tea, avoidance of eggs, dairy, poultry with skin, processed red meat, and saturated fat has been recommended for prostate cancer patients, with no benefit observed from milk thistle, pomegranate, soy, or omega-3 fatty acid supplementation [8]. An inverse association was reported between regular green tea consumption (≥5 cups/day) and breast cancer recurrence for stage I/II patients [9,10]. Regular green tea consumption also reduced the risk of total mortality for the first 60-month post-diagnosis period [11,12].

It seems reasonable to search for diet components to be recommended for breast cancer patients, potentially endangered with cancer recurrence, among plants whose components inhibit the growth of breast cancer cells. There are numerous reports in the literature in this respect, concerning various plants. To mention only a few examples, extracts of *Goniothalamus lanceolatus*, a plant native to the Malaysian rainforest, were reported to inhibit the growth of PEO1 and PEO4 ovary cancer cells, inhibiting cell migration and inducing apoptosis [13]. Gossypol, a polyphenolic aldehyde extracted from the cottonseed, showed an antiproliferative activity against several cell lines, including the SKOV-3 ovary cancer cell line [14]. However, there are good reasons to look for potential diet components to be recommended for such patients among easily available common fruits and vegetables, in which the patients’ diet can be enriched. The aim of this paper was to compare in this respect several fruits and vegetables grown commonly in the temperate and Mediterranean/subtropical climatic zones.

## 2. Materials and Methods

### 2.1. Reagents and Disposables

The flasks (75 cm^2^; cat. no. 156499) and 96-well white plates (cat. no. 165306) were provided by Thermo Fisher Scientific (Waltham, MA, USA). The transparent 96-well culture plates (cat. no 655180), black flat-bottom 96-well plates (cat. no. 655209) and 24-well plates (cat. no. 662160) were obtained from Greiner (Kremsmünster, Austria). Other sterile cell culture materials were provided by Nerbe (Winsen, Germany).

The cell culture medium (McCoy’s 5A (cat. no 22330-021), Roswell Park Memorial Institute (RPMI) medium + GlutaMAX (cat. no 72400-021), Dulbecco’s Modified Eagle Medium (DMEM) + GlutaMAX (cat. no. 21885-025)) and Dulbecco’s phosphate-buffered saline (DPBS) (cat. no. 14040-117) were purchased from Thermo Fisher Scientific (Waltham, MA, USA). The fetal bovine serum (FBS; cat. no. S1813), penicillin–streptomycin solution (cat. no. L0022), trypsin-EDTA solution (10x) (cat. no. X0930) and phosphate-buffered saline without Ca^2+^ and Mg^2+^ (cat. no. P0750) were obtained from Biowest (Nuaillé, France). The 0.33% Neutral Red solution (NR), 0.4% Trypan Blue solution (cat. no. T8154), N-ethylmaleimide (NEM) (cat. no. E3876), trichloroacetic acid (TCA) (cat. no. T4885), diethylenetriaminepentaacetic acid (DTPA) (cat. no. D1133), L-ascorbic acid (cat. no. A0278), gallic acid (cat. no. G7384), 2′,7′-dichlorofluorescein diacetate (H_2_DCFDA) (cat. no. 35845), dihydroethidium (DHE) (cat. no. 37291), dimethyl sulfoxide (DMSO) (cat. no. D2438) and *o*-phtaldialdehyde (OPA) (cat. no. P1378) were provided by Merck (Poznań, Poland). The ethanol (96%; cat. no. 396420113) and glacial acetic acid (cat. no. 568760114), as well as methanol (cat. no. 6219900110) were obtained from Avantor Performance Materials, (Gliwice, Poland). The Mitotracker Deep Red FM (cat. no. M22426) was purchased from Thermo Fisher Scientific (Waltham, MA, USA). The CellTiter-Glo^®^Luminescent Cell Viability Assay (cat. no. G7571) was obtained from Promega (Madison, WI, USA). The JC-1 Mitochondrial Membrane Potential Assay Kit was purchased from Abnova (Taiwan, China). The absorptiometric, fluorometric and luminescence measurements were executed in a Spark multimode microplate reader (Tecan Group LTD., Männedorf, Switzerland).

### 2.2. Lyophilizates, Extracts and Infusions

The whole fruits of bilberry *Vaccinium myrtillus* L., peeled apples (fruits *of Malus domestica* Borkh) and apple peels, peeled plums (fruits of *Prunus domestica* L.) and plum peels were homogenized in ethanol (1:2, *w*/*v*), centrifuged and the supernatants were freeze-dried. Whole tomatoes (fruits of *Solanum lycopersicum* L.), white cabbage (*Brassica oleracea* L. var. *capitata*), onion (*Allium cepa* L.), garlic (*Allium sativum* L.), aloe (*Aloe vera* L), turmeric (*Curcuma longa* L.) rhizome and horseradish (*Armoracia rusticana* G. Gaertn. et al.) root were homogenized in phosphate-buffered saline (1:9, *w*/*v*), extracted with shaking for 30 min and centrifuged. Green and black tea (*Camellia sinensis* (L.) Kuntze), and yew (*Taxus baccata* L.) leaves were extracted with boiling water (1:100, *w*/*v*), incubated for 30 min and the infusions were centrifuged. If not used immediately, the supernatants were stored at −80 °C.

### 2.3. Cell Culture

Two human ovary cancer cell lines (SKOV-3 and PEO1) were used. The normal human fibroblasts MRC-5 cell line was employed as the control cells. The human OC cell line SKOV-3 (HTB-77) and human lung normal fibroblast cell line MRC-5 (CCL-171) were obtained from the American Type Culture Collection (ATCC). The PEO1 (10032308) cell line was purchased from the European Collection of Authenticated Cell Cultures (ECACC).

The SKOV-3 cell line is a hypodiploid OC derived from a 64-year-old Caucasian female with an ovarian serous cyst adenocarcinoma. The number of chromosomes is 43, occurring in over 60% of the cells. These adenocarcinoma cells are positive for many antigens generally used to identify epithelial cancer, such as EMA (epithelial membrane antigen), VIM (vimentin) and cytokeratin alike.

The PEO1 cell line was derived from a malignant effusion from the peritoneal ascites of a patient with a poorly differentiated serous adenocarcinoma after treatment with chlorambucil, 5-fluorouracil and cisplatin. This cell line is positive for hormone receptors, such as the estrogen receptor.

The MRC-5 normal human fibroblasts line was derived from the normal lung tissue of a 14-week-old male fetus. This is a normal diploid human cell line with 46 XY karyotypes.

The SKOV-3 cells were cultured in McCoy’s 5A medium, the PEO1 cells were cultured in RPMI + GlutaMAX and the MRC-5 cells were cultured in DMEM + GlutaMAX. The media used in the experiments were supplemented with 1% *v*/*v* penicillin/streptomycin solution and 10% heat inactivated FBS. The cells were incubated at 37 °C under 5% carbon dioxide and 95% humidity. The cells were passaged at about 85% confluence. The cell viability was estimated by the Trypan Blue exclusion test. The cells were counted in a Thoma hemocytometer (Superior Marienfeld, Lauda-Königshofen, Germany).

### 2.4. Estimation of Cytotoxicity 

The lyophilizates were dissolved in appropriate culture medium to the indicated concentrations. The extracts and infusions were added to the appropriate culture media at indicated proportions. The media were then sterilized by filtration through 0.22 µm filters. The cells were seeded in the wells of a 96-well plate at a density of 1 × 10^4^ cells/well (SKOV-3), 1.5 × 10^4^ cells/well (PEO1) or 7.5 × 10^3^ cells/well (MRC-5 cells). After 24 h, the cell medium was removed, replaced with 100 µL of lyophilizate/extract/infusion-containing medium and the cells were cultivated for 24 h. The cells added with medium containing no additives served as the controls. Then, the medium was removed and replaced with 100 µL of 2% Neutral Red solution, and the cells were incubated at 37 °C for 1 h, washed with PBS; fixed with 100 µL/well of 50% ethanol, 49% H_2_O and 1% glacial acetic acid and shaken (700 rpm) at room temperature for 20 min. The absorbance was measured at 540 nm against 620 nm. The assay was performed in sextuplicate.

### 2.5. Other Assays

The levels of ATP, glutathione and reactive oxygen species (ROS) and protein, mitochondrial mass and changes in the mitochondrial potential were estimated, as previously described in detail [15]. These parameters were estimated after 24 h culture in the presence of extracts or infusions. The polyphenol content was estimated with the Folin–Ciocalteu reagent [16], using gallic acid as a standard and expressed in gallic acid equivalents (GAE).

### 2.6. Statistical Analysis

To estimate the differences between the cells treated with extracts or infusions and the nontreated control, one-way ANOVA with the Least Significant Difference (LSD) post-hoc test (*n* ≥ 3 independent experiments) or the Kruskal–Wallis test (*n* ≥ 6 independent experiments) were performed. A statistical analysis of the data was performed, using the STATISTICA software package (version 13.1, StatSoft Inc., 2016, Tulsa, OK, USA).

## 3. Results

The effects were studied of the lyophilizates, extracts or infusions of several common fruits, vegetables, tea and herb infusions on the proliferation of two lines of human ovary cancer cells and normal human fibroblasts as a control cell line. The lyophilizates of bilberry, peeled apples, apple peels and peeled plums (100–1000 µg/100 µL medium) did not affect the proliferation of any cell line studied. The extract of plum peels (100–1000 µg/100 µL medium) increased significantly the proliferation of SKOV-3 cells, by about 40% (not shown).

**Figure 1:**
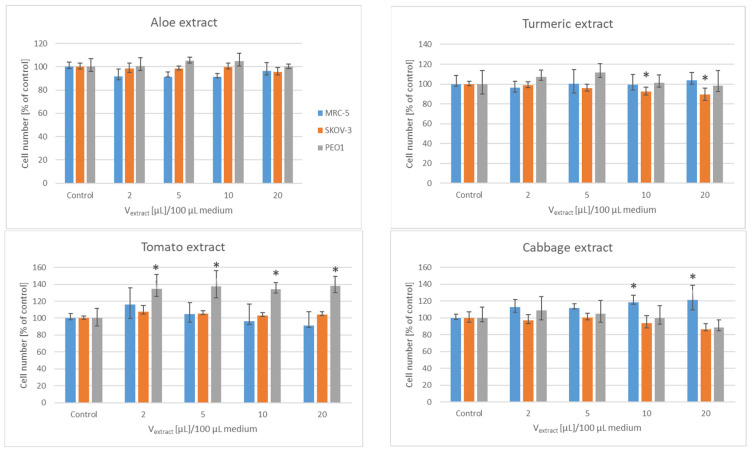

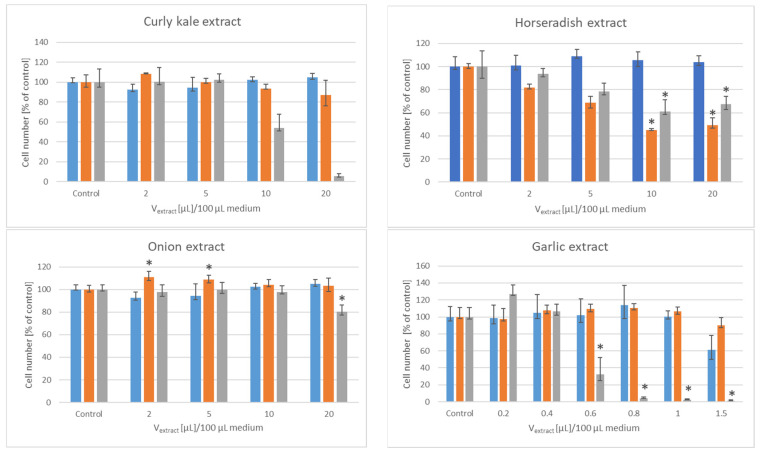
Effect of vegetable extracts on the proliferation of human ovary cancer cells and MRC-5 fibroblasts, estimated with Neutral Red; * *p* < 0.05.

The aloe extract (2–20 µL/100 µL medium, corresponding to a supernatant from 0.2–2 µg aloe/100 µL medium) did not affect the proliferation of the cells of any line studied. The tomato extract did not affect the proliferation of SKOV-3 and MRC-5 cells, but its higher volumes enhanced the proliferation of PEO1 cells. Higher volumes of the turmeric rhizome extract (10 and 20 µL/100 µL medium, corresponding to supernatants from 1 and 2 µg rhizome/100 µL medium) decreased slightly the proliferation of the SKOV-3 cells. Higher volumes of the cabbage extract slightly increased the proliferation of the MRC-5 cells, not affecting significantly the proliferation of the ovary cancer cell lines. Higher volumes of the curly kale extract decreased considerably the proliferation of the PEO1 cells. Higher volumes (10 and 20 µL/100 µL medium, corresponding to supernatants from 1 and 2 µg root/100 µL medium) of the horseradish extract hampered significantly the proliferation of the SKOV-3 and PEO1 cells, not affecting that of the MRC-5 cells. Lower volumes of the onion extract (2 and 5 µL/100 µL medium, i.e., supernatants from 0.2 and 0.5 µg onion/100 µL medium) slightly stimulated the proliferation of the SKOV-3 cells while the volume of (20 µL/100 µL medium i.e., a supernatant from 2 µg onion/100 µL medium) hampered the proliferation of the PEO1 cells. The garlic extract had a dramatic effect on the proliferation of the ovary cancer cells, not affecting significantly the proliferation of the MRC-5 fibroblasts. The proliferation of the PEO1 cells was practically totally inhibited starting from the extract volume of 2 µL/100 µL (a supernatant from 0.2 µg garlic/100 µL medium), while that of the SKOV-3 cells was inhibited starting from the volume of 10 µL/100 µL medium (a supernatant from 1 µg garlic/100 µL medium Figure 1).

**Figure 2:**
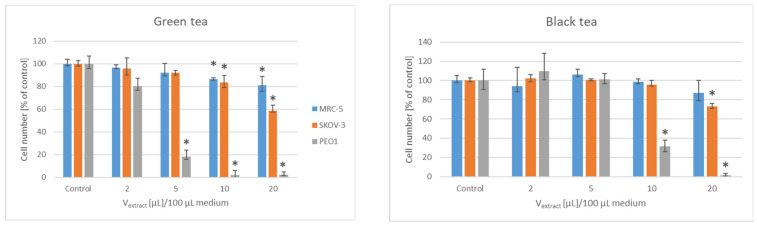

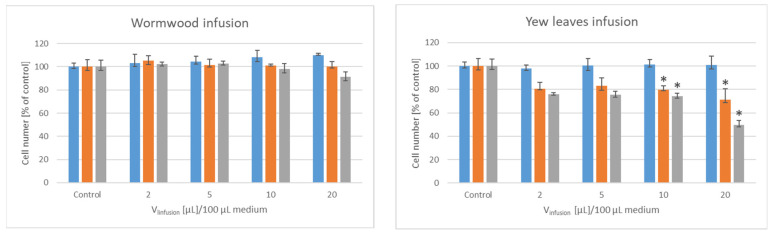
Effect of green and black tea, and of wormwood and yew leaves extract on the proliferation of SKOV-3, PEO1 and MRC-5 cells, estimated with Neutral Red; * *p* < 0.05.

The green tea strongly inhibited the proliferation of the PEO1 cells (starting from the volume of 5 µL infusion/100 µL medium corresponding to infusion from 50 ng tea/100 µL medium); higher volumes of the infusion (10 and 20 µL infusion i.e., infusion from 100 and 200 ng tea/100 µL medium) hampered the proliferation of the SKOV-3 but also the MRC-5 cells. The black tea was somewhat less efficient than the green tea: the proliferation of the PEO1 cells was inhibited by 10 and 20 µL infusion/100 µL medium corresponding to infusion from 100 and 200 ng tea, respectively, while the proliferation of the SKOV-3 cells was moderately inhibited only by 20 µL infusion/100 µL medium. The wormwood extract in the volume range applied (2–20 µL infusion/100 µL medium i.e., infusions from 20–200 ng dry wormwood/100 µL medium) did not significantly affect the proliferation of the cells of any line studied. The higher volumes (10 and 20 µL infusion/100 µL medium i.e., infusions from 100 and 200 ng of leaves/100 µL medium) of the yew leaves extract hampered the proliferation of the SKOV-3 and PEO1 cells, but did not affect the proliferation of MRC-5 cells (Figure 2).

The values of the extract/infusion volumes inhibiting the proliferation of the cells studied in 50% (IV_50_) and the corresponding amounts of the initial materials are listed in Table 1. The presented data allow the conclusion that the extracts of curly kale, horseradish and garlic as well as green and black tea and infusions of yew leaves had the highest potential to inhibit the proliferation of human ovary cells in vitro.

**Figure 3:**
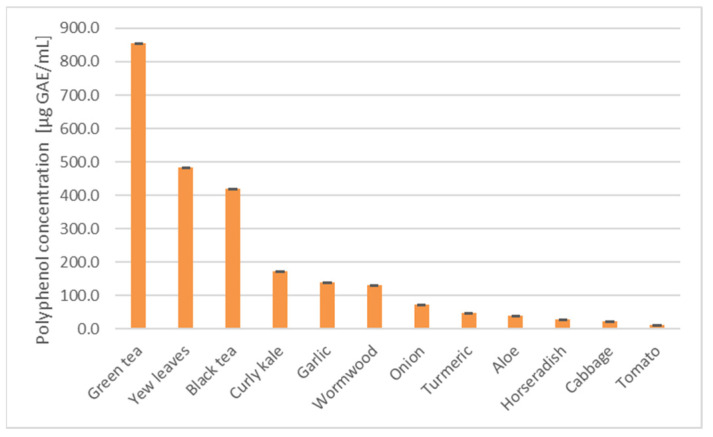
Polyphenol concentration in the extracts and infusions.

The concentration of the total polyphenols (Figure 3) in the extracts and infusions showed a moderate correlation with the inverse of IV_50_ for the most sensitive PEO1 cells (the Pearson correlation coefficient r = 0.23), but when the garlic extract was omitted from the correlation plot, the correlation was much higher (r = 0.88, *p* < 0.05). The omission of the garlic and horseradish extracts increased the value of r to 0.91 (*p* < 0.05).

In order to get an insight into the mechanism of the observed effects, we studied the effects of these three most effective extracts on the ATP level, glutathione content, the level of reactive oxygen species, the mitochondrial mass and the mitochondrial potential of the cells, using extract volumes corresponding to the IV_50_ of the appropriate cell line.

**Figure 4:**
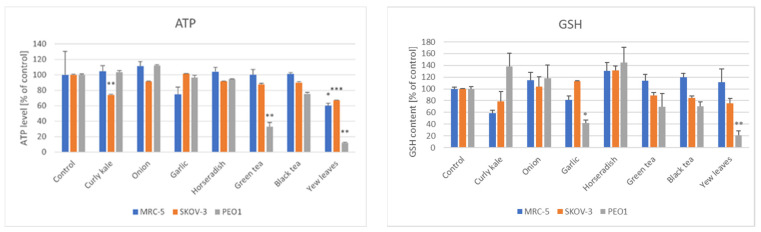
Effect of vegetable extracts, green tea and yew leaves extracts on the ATP level and glutathione (GSH) content of ovary cancer cells and MRC-5 fibroblasts. * *p* < 0.05, ** *p* < 0.001, *** *p* < 0.001 (with respect to Control).

The ATP level was not affected significantly by the majority of the extracts. Only the curly kale extract decreased the ATP level in the SKOV-3 cells and the yew leaves extracts decreased the ATP level in the cells of all of the lines studied, the most dramatic increase being observed in the PEO1 cells. A statistically significant decrease in the GSH content was observed only in the PEO1 cells treated with the garlic and yew leaves extracts (Figure 4).

**Figure 5:**
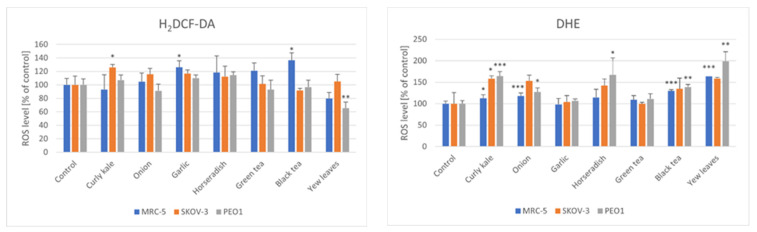
Effect of vegetable extracts and infusions on the level of reactive oxygen species in PEO1, SKOV-3 and MRC-5 cells, estimated with 2′,7′-dichlorodihydrofluorescein diacetate (H_2_DCF-DA) and dihydroethidium (DHE); * *p* < 0.05, ** *p* < 0.001, *** *p* < 0.001 (with respect to Control).

All of the extracts but the yew leaves extract generally tended to increase the ROS levels estimated with H_2_DCF-DA in all of the cell types studied. Statistically significant increases were found for the garlic extract and black tea in the case of the MRC-5 cells, and the curly kale extracts in the case of the SKOV-3 cells. The yew leaves extract decreased the ROS level in the PEO1 cells. The DHE probe, more specific for superoxide, revealed a significant elevation of the ROS levels in the cells of all of the lines studied treated with curly kale, in the MRC-5 and PEO1 cells treated with onion extracts, black tea and yew leaves infusions and in the PEO1 cells treated with the horseradish extract (Figure 5).

The changes induced in the mitochondrial mass did not follow a definite pattern. The curly kale extract increased the mitochondrial mass in the cells of all of the lines studied, the garlic extract decreased the mitochondrial mass in the PEO1 cells, while the horseradish extract decreased the mitochondrial mass in the MRC-5 and SKOV-3 cells increasing it in PEO1 cells.

**Figure 6:**
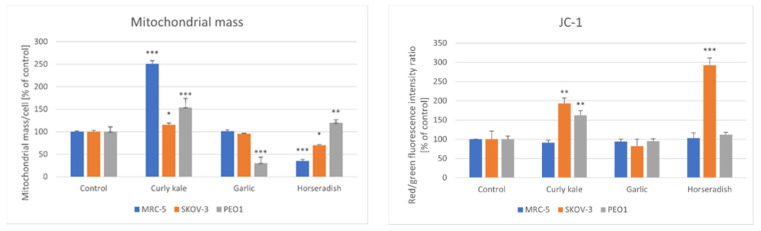
Effect of vegetable extracts on the mitochondrial mass and JC1 red/green fluorescence ratio as a measure of mitochondrial potential in PEO1, SKOV-3 and MRC-5 cells; * *p* < 0.05, ** *p* < 0.001, *** *p* < 0.001 (with respect to Control).

In the MRC-5 cells, the curly kale extract increased while the horseradish extract decreased the mitochondrial mass. The mitochondrial mass was increased by the curly kale, which increased the mitochondrial potential in the SKOV-3 and PEO1 cells; the mitochondrial mass was decreased by the garlic extract in the PEO1 cells, and decreased in the SKOV-3 cells by the horseradish extract. All of the extracts studied did not affect the mitochondrial membrane potential in the MRC-5 cells, apart from the curly kale extract, which induced an increase in the mitochondrial membrane potential in the SKOV-3 and MRC-5 cells, while the horseradish extract augmented the mitochondrial membrane potential in SKOV-3 cells (Figure 6).

## 4. Discussion

All of the plant materials used in this study have been reported to inhibit the growth of cancer cells, though the effects on ovary cancer cells have not been reported in all cases, to our best knowledge. The bilberry anthocyanins were reported to inhibit the growth of several lines of cancer cells [17]. The hydroethanolic and methanolic extract of bilberry fruits inhibited the growth of murine malignant melanoma [18,19]. Apple polyphenols, flavonoids, phloretin and polysaccharides were reported to alleviate colorectal cancer endpoints. The phloretin from apples may be an important anticancer phytochemical based on the results obtained in experiments on the breast, prostate, cervical, lung, esophageal, gastric and blood cancers [20]. The cell viability and proliferation of colon cancers were significantly reduced by plum extracts [21]. The extract obtained from the plum pulp reduced the in vitro proliferation and viability of the A375 melanoma cells [22]. As the fruit peels have a higher content of secondary metabolites, such as polyphenols, than the pulp [23,24], the effects of the pulp and peel extracts of apples and plums were studied separately. However, no growth inhibition of ovary cancer cells was seen with the peel extracts.

Numerous studies demonstrated the anticancer action of turmeric and its main component, curcumin, including ovary cancer [25]. Turmeric extracts (0.4 mg/mL) were found to be cytotoxic to Chinese hamster ovary cells, lymphocytes and Dalton’s lymphoma cells [26]. Tomatoes and tomato products were found to have anticancer activity, especially against prostate cancer, and lycopene was identified as the main active compound [27]. Naringenin, present for example in tomatoes, was effective in the treatment of various malignancies, including breast cancer [28]. The extract of aloe was cytotoxic to human cancer cells, including breast cancer cells [29,30]. Cabbage and sauerkraut consumption was found to correlate negatively with the risk of breast cancer [31]. The cabbage juice inhibited the proliferation of breast cancer cells, showing a preferential activity against breast cancer cells compared with other mammalian cell lines [32]. The extracts of the curly kale exerted an antiproliferative effect on the human colon cancer cell lines [33]. The constituents of horseradish inhibited the proliferation of colon cancer and breast cancer cells [34,35]. It was found that a diet rich in garlic and onions may lower the risk of breast cancer [36,37,38]. The onion extracts were reported to inhibit the growth and induce apoptosis of breast cancer cells [39,40]. Similarly, garlic and garlic-derived compounds reduced the development of mammary cancer in animals and suppressed the growth of human breast cancer cells in culture [41], and garlic extract was demonstrated to prevent the malignant evolution of non-invasive breast tumor cells induced by moderate hypoxia [42].

The anticancer properties of black and especially green tea are well documented. Epidemiological studies revealed that green tea consumption may decrease the incidence of breast cancer [43,44,45], while the conclusions for the black tea were more equivocal [46,47]. Green tea was demonstrated to be cytotoxic for breast cancer cells and inhibit their invasiveness [48,49]. Black tea and its components were also reported to be cytotoxic for ovary cancer cells [50,51]. Yew leaves extract was reported to be cytotoxic for cancer cells [52,53].

In this study, the aqueous extracts of the vegetables were used deliberately, in order to mimic the physiological situation, in which components of the extracts can be ingested in the digestive tract. So, the obtained extracts might contain lower concentrations of active components, which determined the proliferation of cancer cells in previous studies. However, the aim of this study was to compare the extracts obtained under conditions close to the physiological function. The lyophilizates of ethanol extracts of common fruits and extracts of several vegetables did not affect significantly the proliferation of the cells of two ovarian cancer lines in vitro. However, extracts of curly kale, horseradish and garlic inhibited the growth of the SKOV-3 and, especially, PEO1 cells. Moreover, the cytotoxic activity of green and black tea, and of yew leaves extract, were confirmed.

The use of the MRC-5 fibroblasts as control cells is a limitation of this study; normal human ovary cells would be a more appropriate control. However, the fibroblasts are representative of other cell types always present in the body, which would be subject to the action of the bioavailable components of the extracts.

The mechanism of action of vegetable extracts is not obvious and may be not the same for various vegetables. A general phenomenon was the trend for an elevation of the level of ROS in the cells, estimated with the fluorogenic probe H_2_DCFDA; however, only in the case of curly kale did this increase exceed the level of statistical significance for the SKOV-3 cells. The yew leaves extract decreased the ROS level in the PEO1 cells. The ROS level, estimated with the DHE probe, which is more specific for superoxide, especially under the measurement conditions applied [54], pointed to a general increase in the ROS level caused by the extracts and infusions, except for garlic and green tea. Only some of the extracts decreased the ATP level of the cells. Generally, the extracts did not depolarize the inner mitochondrial membrane and either increased or decreased the mitochondrial mass, depending on the extract and cell line studied.

Apparently, the mechanisms of the cytotoxic action of various extracts and infusions can be different as they may be conditioned by various compounds. The cytotoxic effects of tea are mainly attributed to the catechins and theaflavins [51,55]. The cytotoxic action of the yew leaves extracts could be due to the taxanes present in the extracts [56,57]. The correlation observed between the polyphenol content and the inverse IV_50_ observed for most of the extracts and infusions (Figure 3) suggests that the polyphenols may significantly contribute to their effects in these cases.

The anticancer properties of the Brassicaceae family, which includes both the curly kale and horseradish, are ascribed mainly to brassinosteroids [58,59] and isothiocyanates, released from glucosinolates by myrosinase upon plant damage [60,61]. The horseradish extract is also rich in peroxidase, which may modify the cell membrane of the cancer cells [62]. The anticancer action of garlic is attributed mainly to a range of allyl sulfides and their derivatives [63,64].

The results of this study confirm that green and black tea, and extracts of curly kale, garlic and horseradish, can be considered as potential beverages and vegetables for ovarian cancer patients. Yew leaves are known to be toxic, so their direct use cannot be recommended. Of course, the direct effects of the extracts on the cancer cells are very preliminary results, since the extract components which act on the cells in such experiments may be not easily bioavailable and metabolically modified. e.g., if peroxidase participates in the effects of the horseradish extract, this contribution will not occur in vivo. Nevertheless, these results may provide a clue for further experiments. Moreover, recent techniques of micro- and nano-encapsulation can allow the direct delivery of extract components to the cells. In practical terms, tea and vegetables such as curly kale, garlic and horseradish can at least not be avoided by ovarian cancer patients. An additional argument for the inclusion of, in particular, garlic and horseradish in the diet are the changes in taste during ovarian cancer treatment, mainly due to damage to the taste receptor cells localized on the tongue epithelium and throughout the digestive tract, caused by radiation or chemotherapeutic agents. In order to increase food palatability, the addition of more condiments or enrichment with components of strong taste is recommended [65].

## Figures and Tables

**Table 1:** Inhibitory volumes of extracts of fruits and vegetables, and of herbal infusions (as well as mass of the initial material) per 100 µL medium on the proliferation of ovary cancer cells.

Lyophilizate/ Extract/Infusion	IV_50_ [µL]/Mass of Initial Material PEO1	SKOV-3
Bilberry	ND	ND
Apple	ND	ND
Apple peel	ND	ND
Plum	ND	ND
Plum peel	ND	ND
Aloe	ND	ND
Turmeric	ND	ND
Tomato	ND	ND
Cabbage	ND	ND
Curly kale	10.6	ND
Horseradish	14.2	ND
Onion	67.4 (6.74 μg) ^E^	ND
Garlic	1.1 (111 ng)	3.3 (333 ng)
Green tea	3.3 (33.3 ng)	25.3 (253 ng) ^E^
Black tea	6.5 (65 ng)	21.1 (211 ng)
Wormwood	ND	ND
Yew leaves	19.9 (199 ng)	275.3 (2.753 mg) ^E^

ND—could not be determined on the basis of obtained data (from up to 20 µL of the extract); ^E^—extrapolated. For selected extracts, growth inhibition was studied in an extended volume range. MRC-5 fibroblasts: ND in all cases.

## Data Availability

Data available from the corresponding author upon reasonable request.
